# Supplementary material for: Rational design of pyrrole derivatives with aggregation-induced phosphorescence characteristics for time-resolved and two-photon luminescence imaging
Source: Nat Commun. 2021 Aug 12;12:4883. doi: 10.1038/s41467-021-25174-6 (PMC8361132; doi:10.1038/s41467-021-25174-6)
Supplement: Supplementary file 1 — Supplementary Information [file 41467_2021_25174_MOESM1_ESM.pdf]

Rational Design of Pyrrole Derivatives with Aggregation-Induced Phosphorescence Characteristic for Time-Resolved and Two-Photon Luminescence Imaging

Jianhui Yang#, Yahui Zhang#, Xinghui Wu, Wenbo Dai, Dan Chen, Jianbing Shi, Bin Tong, Qian Peng, Haiyan Xie, Zhengxu Cai*, Yuping Dong, Xin Zhang*

1. Materials and characterization: 2
2. Cell culture： 3
3. In vivo imaging: 4
4. Absorption spectra and emission spectra of TePP and PPP: 5
5. Photophysical properties of TPM and TPM-Cl: 5
6. Morphology characterizations of nanoparticles: 8
7. Single crystal data and time-dependent density functional theory (TD-DFT) calculations: 11
8. Cell viabilities and intensity of lifetime decay profile: 15
9. High resolution mass spectrometry, NMR spectra and HPLC spectra: 16

1. Materials and characterization:

Benzoyl chloride derivatives, triethylamine, and 4-dimethylaminopyridine were purchased from Energy Chemical. Fetal bovine serum (FBS), 0.25% trypsinethylenediaminetetraacetic acid (EDTA), Dulbecco's modified eagle's medium (DMEM) and penicillin-streptomycin were obtained from Gibco. PBS and trpsin were purchased from Coring Co., Ltd. DiD was bought from Thermo Fisher Scientifics. MTS were purchased from Beyotime technologies Co., Ltd. The deionized water was used for all experiments.

Molecular structures of TPM/TPM-Cl were confirmed by Nuclear Magnetic Resonance (NMR). ^{1}H and ^{13}C NMR spectra were carried out by Bruker ARX400 spectrometer with $CDCl_3$ as the solvent, and high resolution mass spectrum were performed by using a Thermo Q-Extractive. High performance liquid chromatography (HPLC) was performed on Thermo Fisher Vanquish Series. Column: Accucore Vanquish C18+ 100 x 2.1 mm. Particle size:1.5 μm. Mobile phase: acetonitrile. Flow rate: 0.2 mL/min. Column oven 40℃. The single crystal X-ray diffraction data collections were carried out on a Rigaku AFC-10/Saturn 724+CCD diffractometer with graphite-monochromated Mo Kα radiation (λ=0.71073 Å) using the multi-scan technique. The structures were determined and refined by using SHELXL-2015 and Olex2 software. UV-Vis absorption spectra were measured by Persee TU-1901 in solution and Shimadzu UV-2600 in solid state. XRD was recorded using an Rigaku Smartlab SE. TEM was detemined by JEM2100f. Fluorescence spectra were recorded by a Hitachi F-7000 spectrophotometer. Phosphorescence spectra were measured by using FLS920 lifetime and steady state spectrometer. Solid-state emission quantum yields (Φ) were collected by using FluoroMax-4 (Horiba Jobin Yvon) fluorimeter equipped with integrated sphere. Cell imaging was performed on a Nikon, Japan laser scanning confocal microscope. Two-photon imaging and phosphorescence lifetime imaging were performed on Q2 ISS and Horiba.

TD-DFT calculations were performed on Gaussian 09 program (Revision D. 01). The ground-state (S_0) geometries were optimized with B3LYP by using 6-311G(d) basis sets. The excitation energies in the n-th singlet (S_n) and n-th triplet (T_n) states were obtained by using the TD-DFT method based on single-crystal an optimized molecular structure at ground state. The transition orbital compositions were

obtained to evaluate the possible singlet-triplet intersystem crossings (ISC) channels from S_1 to T_n. We further calculated the SOC matrix elements between S_1 and T_n states with ORCA program (Version 4. 2. 1).

2. Cell culture:

Cell Culture: HeLa cells were obtained from National Infrastructure of cell line Resource (NICR), cultured with Dulbecco's modified eagle medium (DMEM) supplemented with 10% fetal bovine serum (FBS), 100 U/mL penicillin, and 100 mg/mL streptomycin at 37 °C in a humidified atmosphere of 5% CO_2.

In Vitro Cytotoxicity: HeLa cells (1×10^4 cells/well) were seeded in 96-well plates. Four hours later, the medium was replaced by 100 μL of fresh medium, and the cells were cultured with TPM at different concentrations for another 24 h. Then the medium was discarded, and the cells were incubated with MTS solutions (5 mg/mL) for 2 h at 37 °C. The absorbance of each well was measured at 490 nm by a microplate reader. Cells without treatment were included as a negative control.

The test of amount diffused in HeLa cells: HeLa cells were cultured overnight in a humidified incubator at 37°C with 5% CO_2. 1.0×10^{-4} mol/L of TPM (100 μL) was added into cells with 900 μL of DMEM. 1.0×10^{-4} mol/L of TPM (100 μL) was added into 900 μL of DMEM without Hela cells as control group. After 10 min incubation, all solutions were taken out and the UV tests were measured.

Confocal fluorescent imaging: HeLa cells were seeded in Φ20 mm glass bottom cell culture dishes ($1.0 \pm 0.05 \times 10^6$ cells in each dish). After overnight culture in a humidified incubator at 37 ^{o}C with 5% CO_2, culture medium was removed and cells were stained with 10 μM of TPM for 10 min in DMSO/DMEM (10% DMSO) solution at 37 ^{o}C. After washed by PBS for 3 times, HeLa cells were fixed with 4% fixative solution for 10 min. Before imaging, each dish was washed by PBS for 3 times. The fluorescence imaging of TPM in cells was recorded with a Leica TCS SP5 laser scanning confocal microscope. For co-localization with LysoTracker, the fixed cells were stained with LysoTracker (5 μm) for 10 min at 37 ^{o}C.

Data analysis: The data of Confocal fluorescent imaging was measured with NIS Viewer 4.50.

Two-photon imaging and phosphorescence lifetime imaging: HeLa cells were seeded in Φ20 mm glass bottom cell culture dishes ($1.0 \pm 0.05 \times 10^6$ cells in each dish). After overnight culture in a humidified incubator at 37 °C with 5% CO_2, culture medium was removed and cells were stained with 10 μM of TPM for 10 min in DMSO/DMEM (10% DMSO) solution at 37 °C. After washed by PBS for 3 times, HeLa cells were fixed with 4% fixative solution for 10 min. Before imaging, each dish was washed by PBS for 3 times. The two-photon imaging and phosphorescence lifetime imaging of TPM in cells was recorded with a Nikon scanning confocal microscope and Q2 (ISS), respectively.

Data analysis: The data of two-photon imaging and phosphorescence lifetime imaging was measured with Image J 1.52.

3. In vivo imaging:

Animals: Six-weeks-old BALB/c nude mice were purchased from the ital River Laboratory Animal Technology Co., Ltd. (Beijing, China).

Statement of ethical approval: All animal studies were performed in accordance with the Regulations for Care and Use of Laboratory Animals and Guideline for Ethical Review of Animals (China, GB/T 35892-2018) and the overall project protocols were approved by the Animal Ethics Committee of Beijing Institute of Technology. The accreditation number is BIT-EC-SCXK(Jing) 2019-0010-M-2020019 promulgated by Animal Ethics Committee of Beijing Institute of Technology.

Feeding conditions all the animals were submitted to controlled temperature conditions (22~26 °C), humidity (50~60%) and light (12 h light/12 h dark, 15~20 LX). They had access to water and food ad libitum in barrier system of Beijing Institute of Technology. (SYXK (Jing) 20170013)

Afterglow subcutaneous imaging: The living mice were anesthetized with Isoflurane. TPM afterglow nanoparticles (47.5 μg mL^{-1}, 100 μL) were subcutaneously injected to the left flank of the living mice. Afterglow images of the living mice were then captured on IVIS Spectrum CT In Vivo Imaging System under bioluminescence mode (open filter; exposure time: 60 s) instantly after irradiation of the mice with LED UV lamp (360~370 nm; 25.5 mW/cm^2) for 60 s.

Data analysis: Afterglow luminescence in afterglow images captured on IVIS Spectrum CT In Vivo Imaging System was quantified by ROI analysis on Living Imaging.

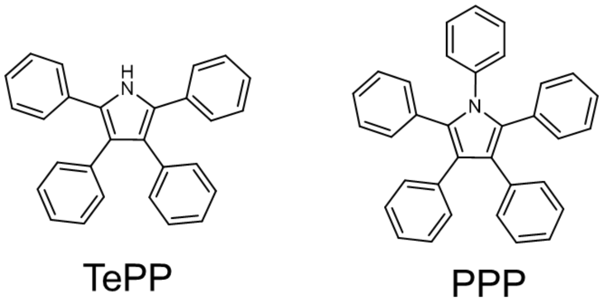

Figure S1. Chemical structures. Chemical structures of tetraphenylpyrrole (TePP) and pentaphenylpyrrole (PPP) molecule.

4. Absorption spectra and emission spectra of TePP and PPP:

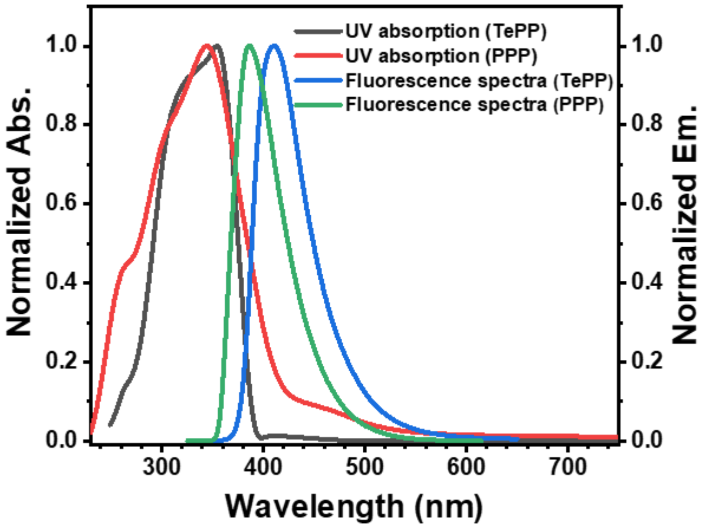

Figure S2. UV absorption spectra and fluorescence spectra of TePP and PPP in solid states at room temperature.

5. Photophysical properties of TPM and TPM-Cl:

Table S1. Photophysical properties of TPM and TPM-Cl.

Samples	THF	f_w (80%)			Solid		
	Abs (nm)	Em (nm)	Φ_{PL} (%)	τ (μs)	Em (nm)	Φ_{PL} (%)	τ (μs)
TPM	249	527	3.50	18.5	528	3.65	20.1
TPM-Cl	259	549	3.61	7.4	544	3.76	8.9

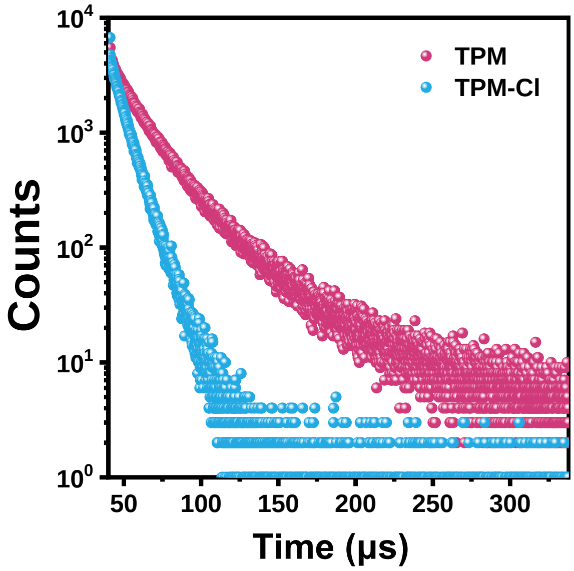

Figure S3. Decay spectra under ambient condition in solid states.

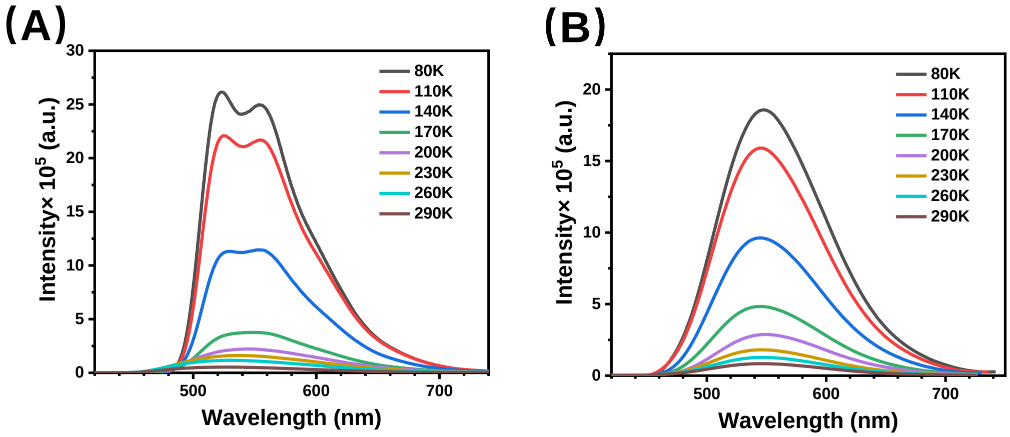

Figure S4. Temperature dependent phosphorescence spectra of TPM (A) and TPM-Cl (B) in solid state.

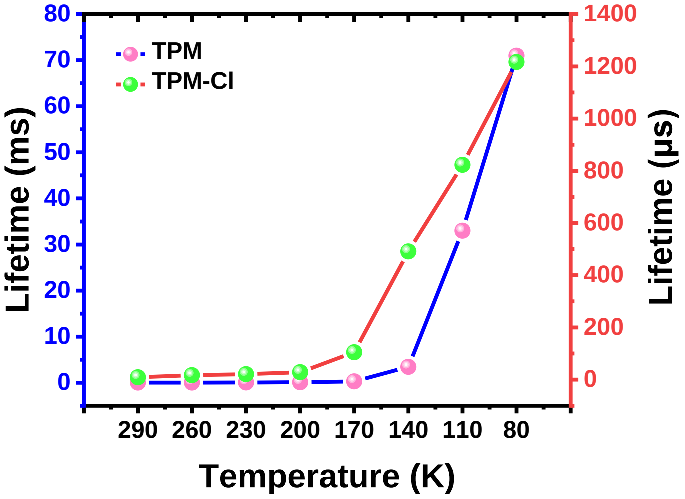

Figure S5. Temperature dependent phosphorescence lifetime of TPM and TPM-Cl in solid state.

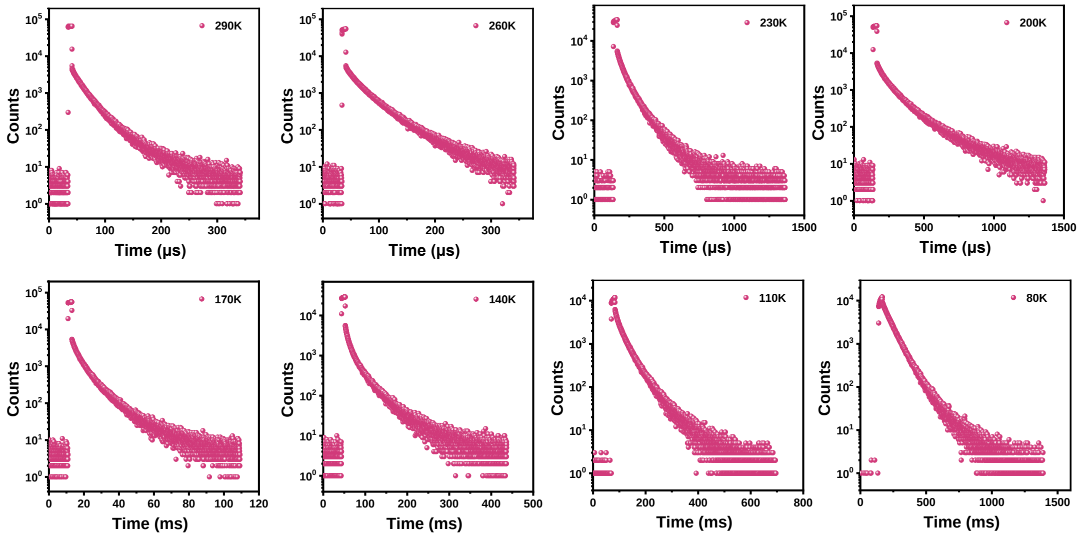

Figure S6. The phosphorescence lifetime of TPM at different temperatures.

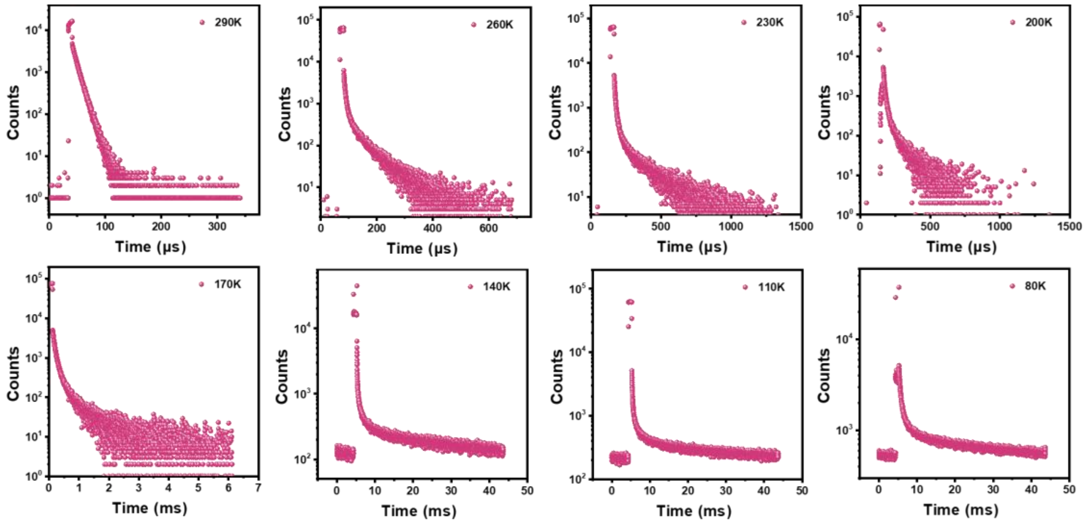

Figure S7. The phosphorescence lifetime of TPM-Cl at different temperatures.

Table S2. Lifetime and quantum yield of TPM at different water fraction in THF/water mixtures.

Water content	0%	30%	50%	60%	80%	90%	95%	99%
Concentration	100 μM	100 μM	100 μM	100 μM	100 μM	100 μM	100 μM	100 μM
τ (μs)	-	-	-	17.3	18.5	16.6	13.2	12.9
Φ_{PL} (%)	-	-	-	2.54	3.50	2.08	1.02	0.99

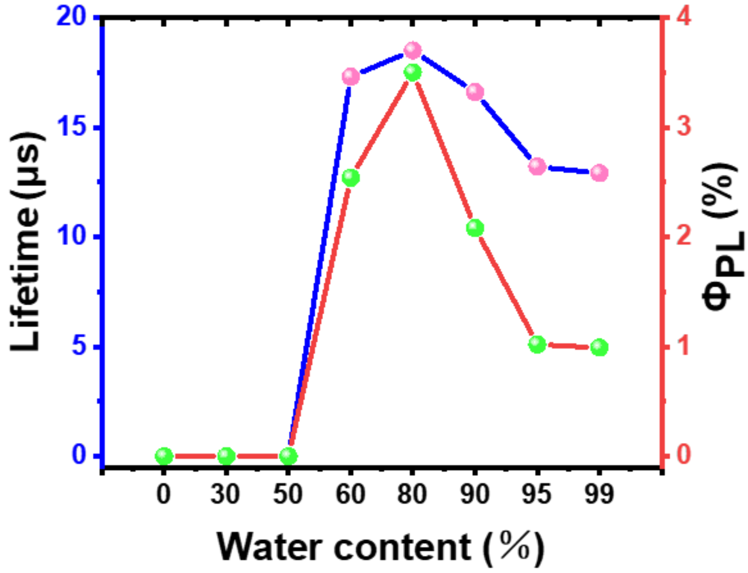

Figure S8. Lifetime and quantum yield of TPM at different water fraction in THF/water mixtures.

6. Morphology characterizations of nanoparticles:

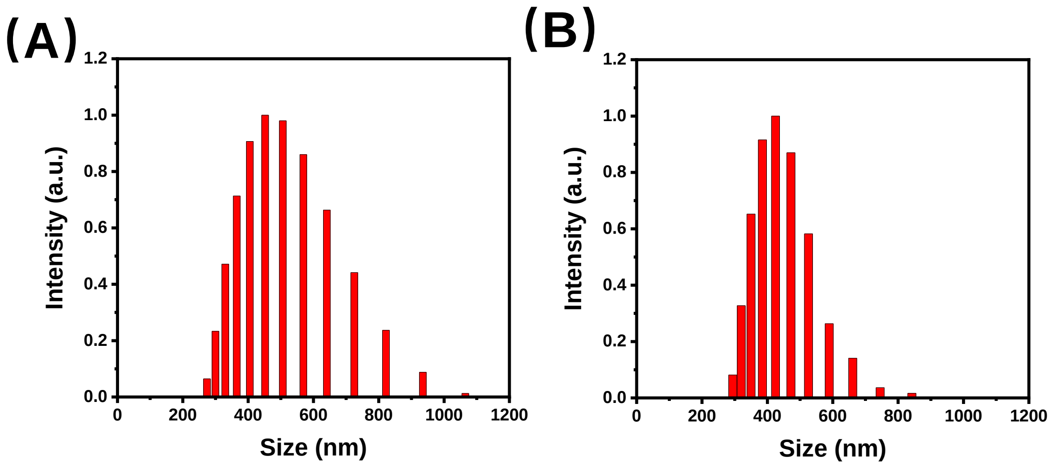

Figure S9. Dynamic light scattering data for TPM (A) and TPM-Cl (B) nanoparticles that obtained from 80% water content in THF/water mixture.

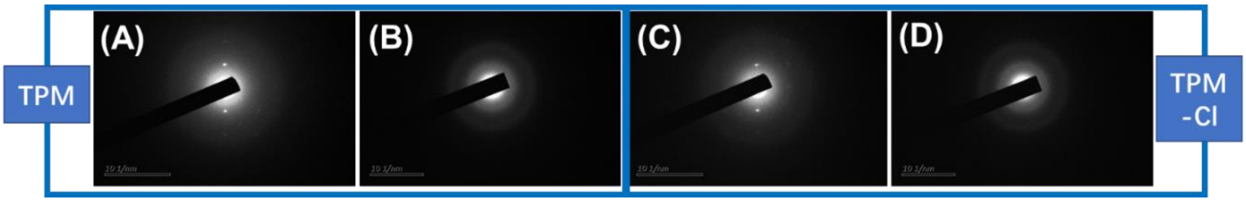

Figure S10. The selected area electron diffraction of TPM/TPM-Cl nanoparticles with 80% (A and C) and 90% (B and D) water fractions, respectively. Each experiment repeated 3 times independently.

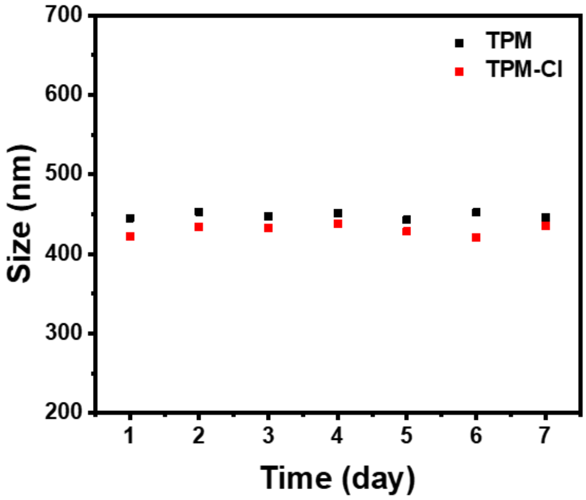

Figure S11. The variation of particle sizes measured by dynamic light scattering (DLS) after storage for 1-7 days at room temperature.

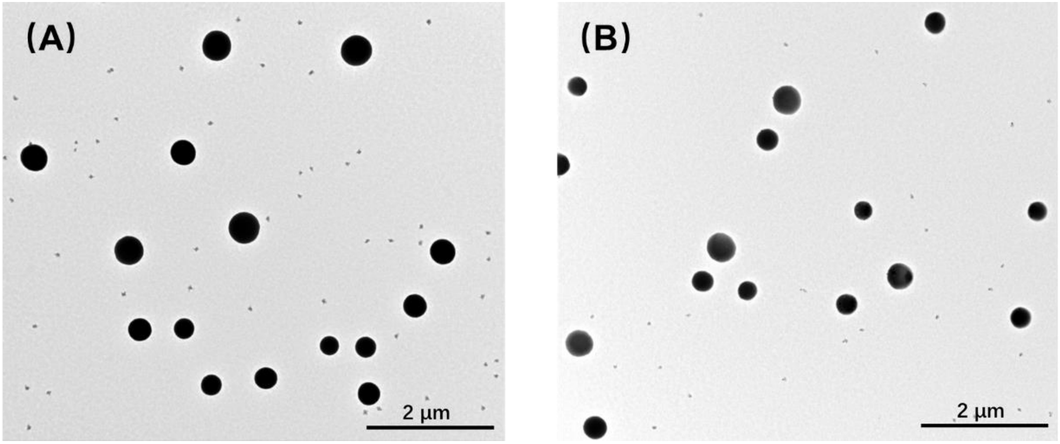

Figure S12. TEM images of (A) TPM and (B) TPM-Cl nanoparticles in the DMSO/PBS mixtures (10 μM, DMSO: PBS = 10: 90, volume ratio). Each experiment repeated 3 times independently.

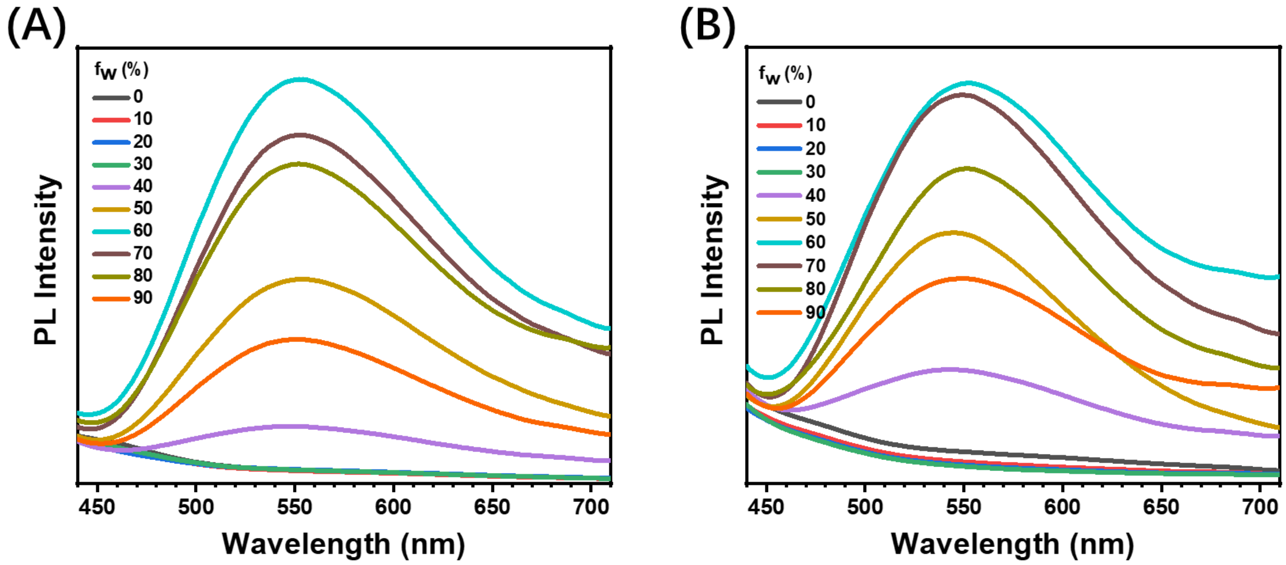

Figure S13. Emission spectra of TPM/TPM-Cl nanoparticles with different water fractions in the DMSO/PBS mixtures.

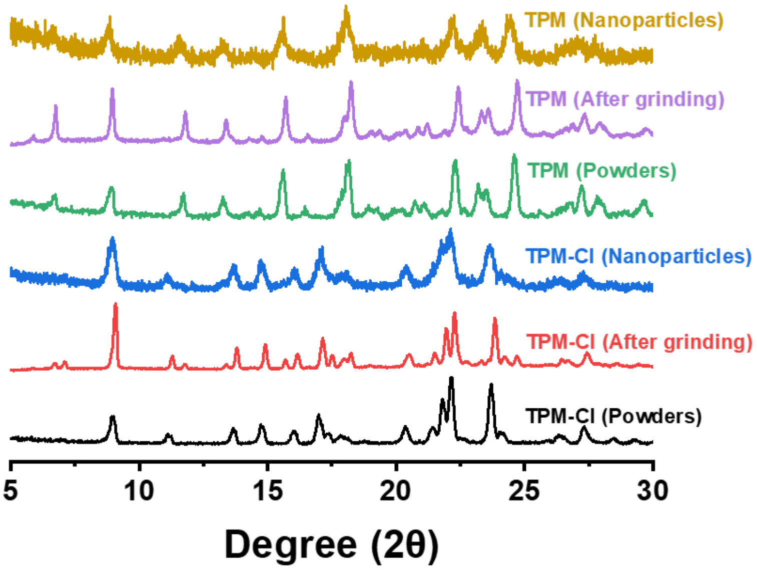

Figure S14. XRD patterns of TPM and TPM-Cl in crystalline powders, grinding powders, and nanoparticles.

The preparation method of nanocrystals used for XRD: Nanocrystals used for XRD were prepared by nanoprecipitation method in THF/water mixture. In detail, a stock solution of TPM/TPM-Cl in THF (10^{-3} M, 1 mL) was injected rapidly into 10 mL water and vigorously stirred at room temperature for 10 min. Then the nanoparticles of TPM/TPM-Cl were dried by freeze drying method for XRD measurements.

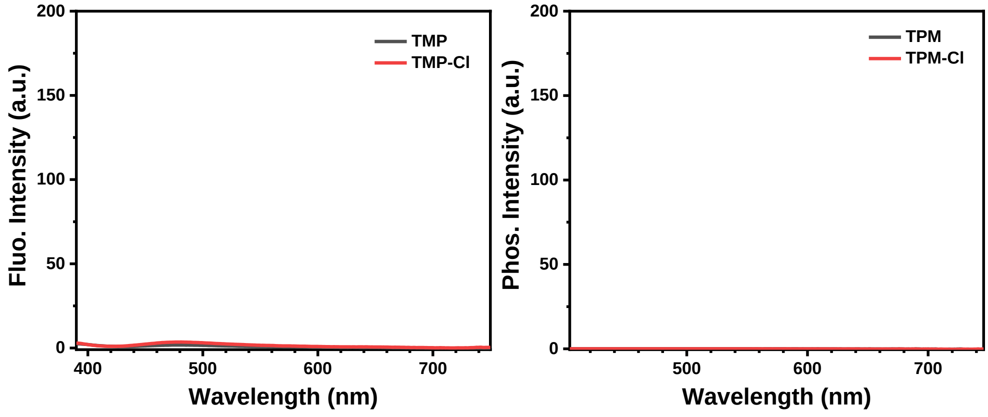

Figure S15. The fluorescence spectra and phosphorescence spectra of TPM and TPM-Cl in PMMA (Mw 120,000, TPM/TPM-Cl: PMMA = 1: 100, mass ratio).

7. Single crystal data and time-dependent density functional theory (TD-DFT) calculations:

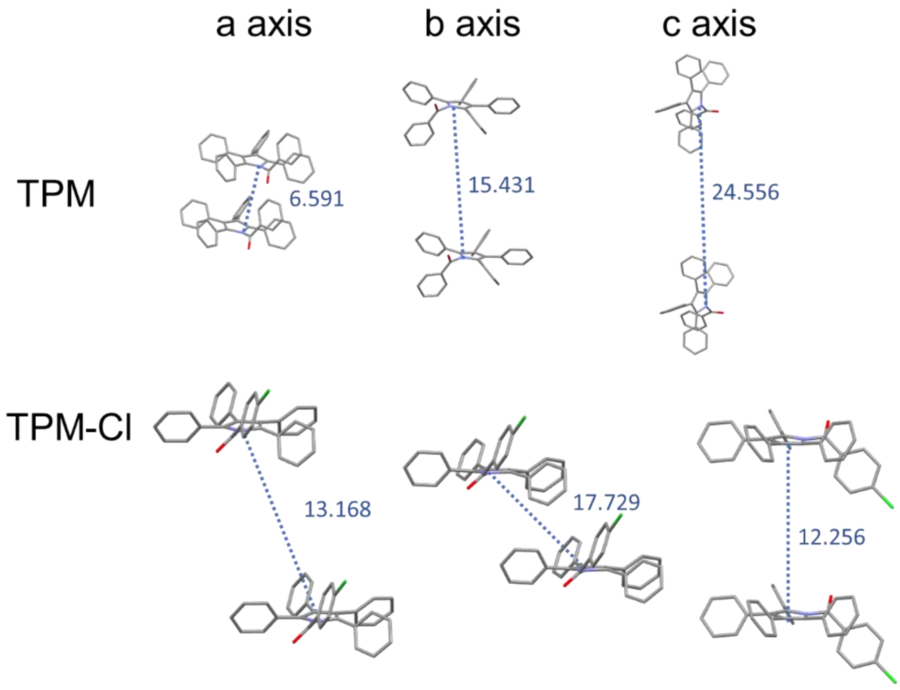

Figure S16. The distance that one TPM/TPM-Cl molecular occupied in the axial direction.

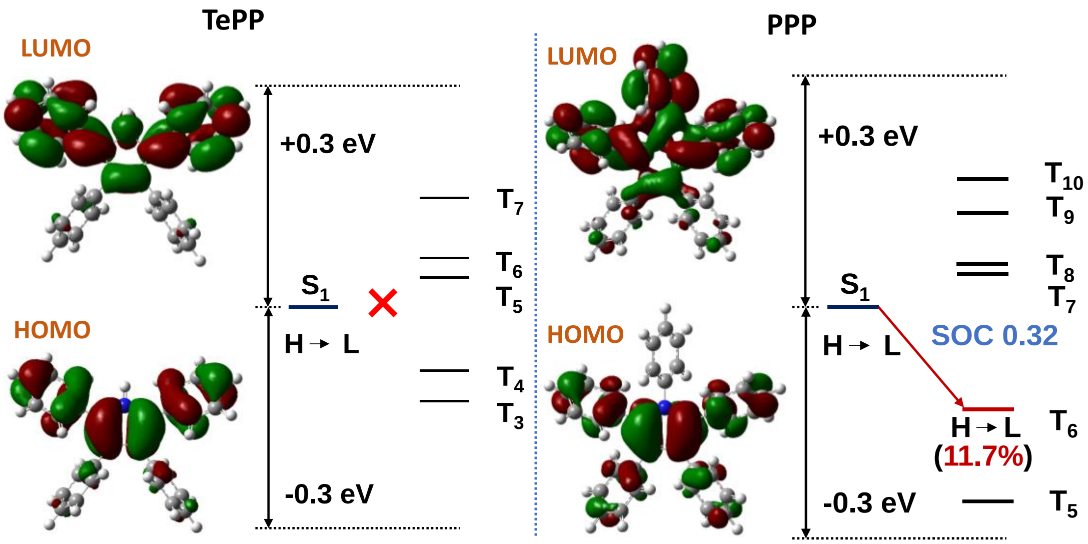

Figure S17. The molecular orbitals of the TePP and PPP.

Table S3. The energy level and energy gap of pyrrole derivatives.

Samples	HOMO (eV)	LUMO (eV)	E_g (eV)
TPM	-5.55	-2.20	3.35
TPM-Cl	-5.64	-2.38	3.26
TePP	-5.31	-1.20	4.11
PPP	-5.42	-0.95	4.47

Table S4. Crystal data for TPM and TPM-Cl.

TPM	
CCDC number	1975269
Empirical formula	$C_{35}H_{25}NO$
Formula weight	475.56
Temperature/K	163.15
Crystal system	monoclinic
Space group	$P2_1/n$
a/Å	6.5912(13)
b/Å	15.431(3)
c/Å	24.556(5)
α/°	90
β/°	90.21(3)
γ/°	90
Volume/Å^3	2497.6(9)
Z	4
ρ_{calc}g/cm^3	1.265
μ/mm^{-1}	0.075
F(000)	1000.0

Crystal size/mm^3	0.13 × 0.1 × 0.06
Radiation	MoKα (λ = 0.71073)
2Θ range for data collection/°	3.118 to 54.908
Index ranges	-8 ≤ h ≤ 8, -19 ≤ k ≤ 19, 0 ≤ l ≤ 31
Reflections collected	5690
Independent reflections	5690 [R_{sigma} = 0.0592]
Data/restraints/parameters	5690/0/335
Goodness-of-fit on F^2	1.464
Final R indexes [I>=2σ (I)]	R_1 = 0.1357, wR_2 = 0.3694
Final R indexes [all data]	R_1 = 0.1474, wR_2 = 0.3826
Largest diff. peak/hole / e Å^{-3}	1.02/-0.54

TPM-Cl	
CCDC number	1975270
Empirical formula	$C_{35}H_{24}ClNO$
Formula weight	510.00
Temperature/K	153.15
Crystal system	monoclinic
Space group	$P2_1/c$
a/Å	13.168(3)
b/Å	17.729(4)
c/Å	12.256(3)
α/°	90
β/°	114.89(3)
γ/°	90
Volume/Å^3	2595.6(11)
Z	4
ρ_{calc}g/cm^3	1.305
μ/mm^{-1}	0.177
F(000)	1064.0
Crystal size/mm^3	0.13 × 0.12 × 0.11
Radiation	MoKα (λ = 0.71073)
2Θ range for data collection/°	4.594 to 54.934
Index ranges	-12 ≤ h ≤ 17, -22 ≤ k ≤ 23, -15 ≤ l ≤ 15
Reflections collected	17620
Independent reflections	5892 [R_{int} = 0.0544, R_{sigma} = 0.0635]
Data/restraints/parameters	5892/0/343
Goodness-of-fit on F^2	1.237
Final R indexes [I>=2σ (I)]	R_1 = 0.0912, wR_2 = 0.1524
Final R indexes [all data]	R_1 = 0.1129, wR_2 = 0.1632
Largest diff. peak/hole / e Å^{-3}	0.24/-0.35

Table S5. The singlet and triplet excited state transition configurations of TPM revealed by TD-DFT calculations. The matched triplet excited states that contain the same orbital transition components of S_1 were highlighted in red.

	States	Energy (eV)	Transition configuration (%)
TPM	S_1	2.6883	H → L 99.0%
	T_1	2.5593	H → L 89.3%, H → L+1 5.9%

Table S6. The singlet and triplet excited state transition configurations of TPM-Cl revealed by TD-DFT calculations. The matched triplet excited states that contain the same orbital transition components of S_1 were highlighted in red.

	States	Energy (eV)	Transition configuration (%)
TPM-Cl	S_1	2.5987	H → L 99.0%
	T_1	2.4800	H → L 91.3%

Table S7. The singlet and triplet excited state transition configurations of TePP revealed by TD-DFT calculations. The matched triplet excited states that contain the same orbital transition components of S_1 were highlighted in red.

	States	Energy (eV)	Transition configuration (%)
TePP	S_1	3.8037	H → L 98.4%
	T_1	2.6905	H → L 89.3%
	T_2	3.3795	H → L+1 52.9%, H-7 → L 10.0%, H → L+6 7.4%, H → L+3 5.2%
	T_3	3.6833	H-1 → L+1 14.4%, H-3 → L+4 12.0%, H-2 → L+5 9.1%, H-1 → L+6 8.7%, H-4 → L+7 8.0%, H-2 → L+2 6.8%
	T_4	3.7121	H-1 → L+4 11.8%, H-2 → L+7 9.8%, H-3 → L+1 8.3%, H-3 → L+6 6.7%, H → L+6 5.6%, H-1 → L+5 5.2%, H-4 → L+5 5.0%
	T_5	3.8837	H → L+2 65.5%, H → L+5 7.8%
	T_6	3.9226	H → L+3 72.5%, H → L+1 8.8%
	T_7	4.0840	H-5 → L+3 13.3%, H-9 → L 11.6%, H → L+5 10.5%, H-6 → L+2 9.0%, H → L+11 5.9%
	T_8	4.1312	H-1 → L 18.4%, H → L+1 15.5%, H-6 → L+3 6.9%, H-7 → L 6.0%, H-8 → L 5.2%

Table S8. The singlet and triplet excited state transition configurations of PPP revealed by TD-DFT calculations. The matched triplet excited states that contain the same orbital transition components of S_1 were highlighted in red.

	States	Energy (eV)	Transition configuration (%)
PPP	S_1	3.9423	H → L 86.1%, H → L+2 12.8%
	T_1	3.0264	H → L 74.6%, H → L+2 5.6%
	T_2	3.4235	H → L+1 28.7%, H → L+3 20.0%, H-1 → L 8.7%
	T_3	3.5990	H → L+6 12.1%, H-1 → L+7 11.4%, H-2 → L+9 6.4%, H-1 → L 6.0%, H-5 → L+3 5.1%
	T_4	3.6130	H-1 → L+3 13.1%, H → L+7 9.1%, H-4 → L+9 7.3%, H-1 → L+8 6.0%, H-1 → L+4 5.6%, H-2 → L+4 5.0%
	T_5	3.6713	H-9 → L+1 16.4%, H-3 → L+2 13.4%, H-3 → L 11.9%, H-11 → L+2 7.0%, H-1 → L+2 6.2%, H-1 → L 5.9%, H-8 → L+2 5.5%
	T_6	3.8096	H → L+2 37.0%, H → L 11.7%
	T_7	4.0362	H → L+2 45.9%, H → L+5 12.7%
	T_8	4.0475	H → L+3 43.3%, H → L+1 30.6%, H → L+4 11.7%
	T_9	4.0935	H → L+1 25.5%, H → L+3 15.4%, H → L+6 8.6%, H-6 → L+5 5.2%, H-4 → L+4 5.1%
	T_{10}	4.1296	H-1→ L 62.2%

8. Cell viabilities and intensity of lifetime decay profile:

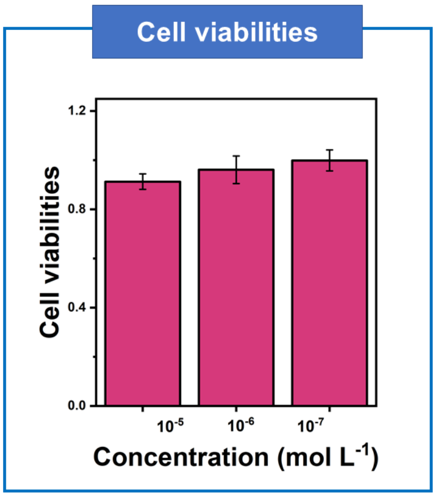

Figure S18. Cell viabilities of HeLa cells in the presence of different concentrations of TPM. All bars represent mean ± SD (n = 3).

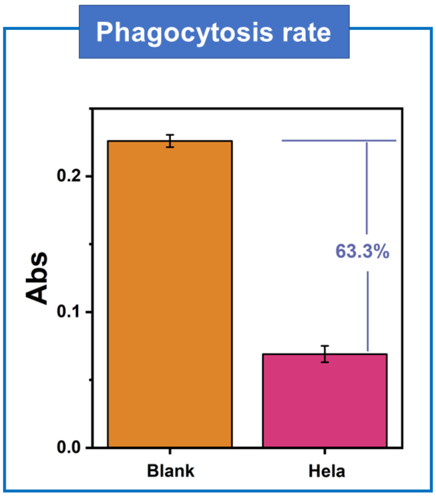

Figure S19. The amount of TPM in HeLa cells after 10 min incubation in the concentration at [TPM] = 10 μM. All bars represent mean ± SD (n = 3).

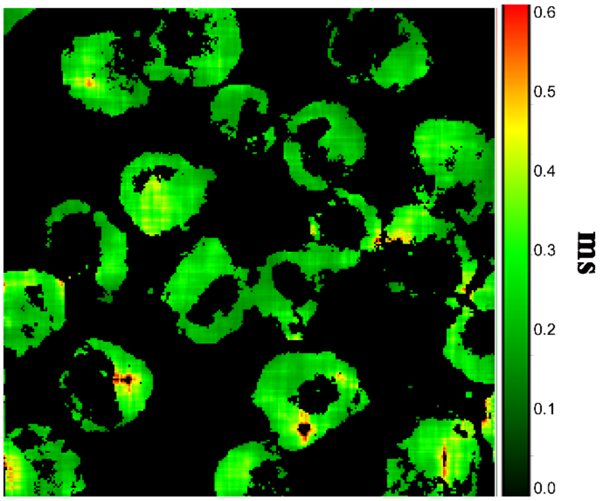

Figure S20. Intensity of lifetime decay profile of emission band in HeLa cell.

9. High resolution mass spectrometry, NMR spectra and HPLC spectra:

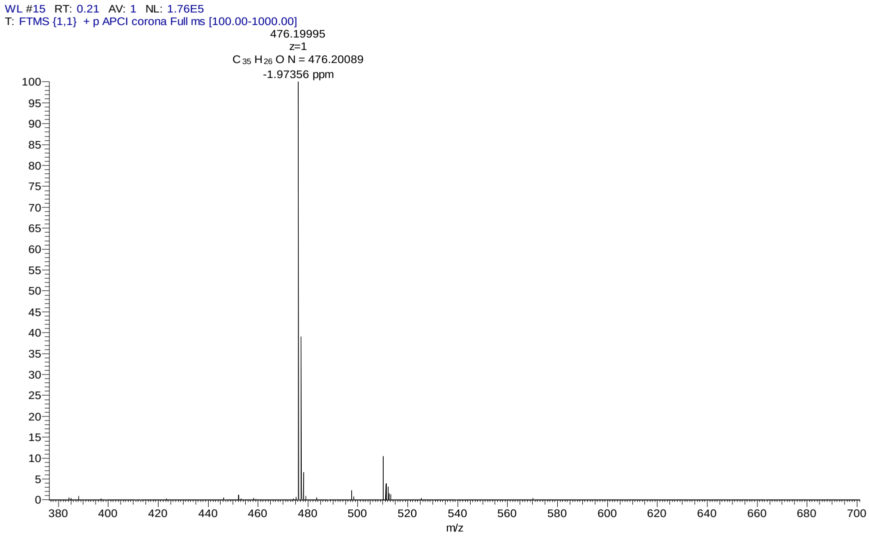

Figure S21. High resolution mass spectrum of TPM.

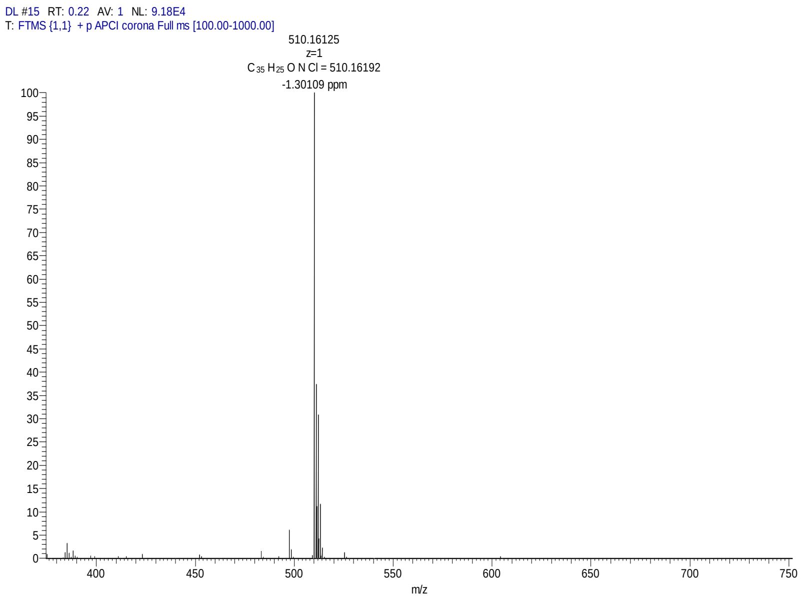

Figure S22. High resolution mass spectrum of TPM-Cl.

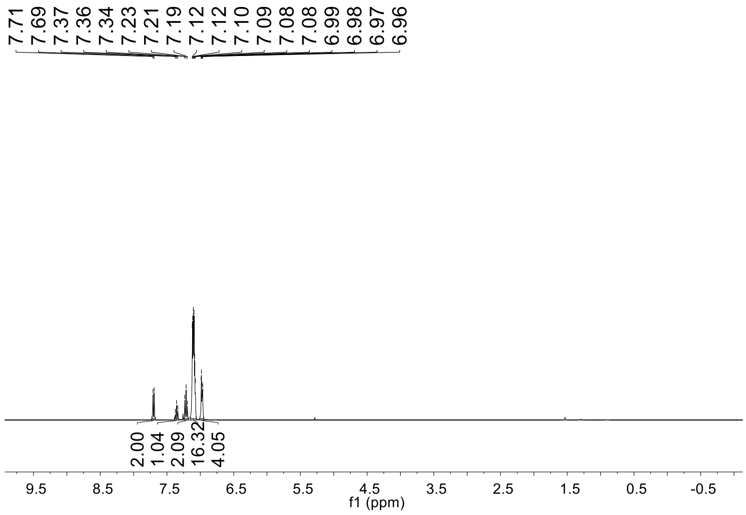

Figure S23. ^{1}H NMR of TPM ($CDCl_3$, 293 K).

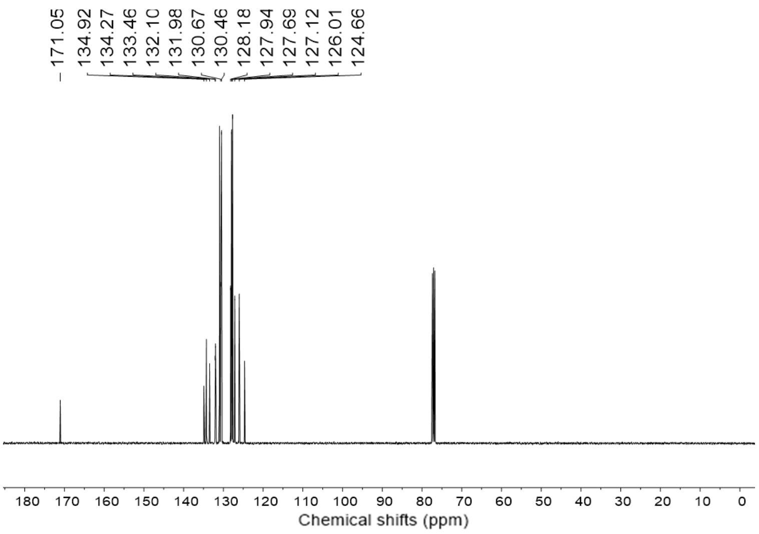

Figure S24. ^{13}C NMR of TPM ($CDCl_3$, 293 K).

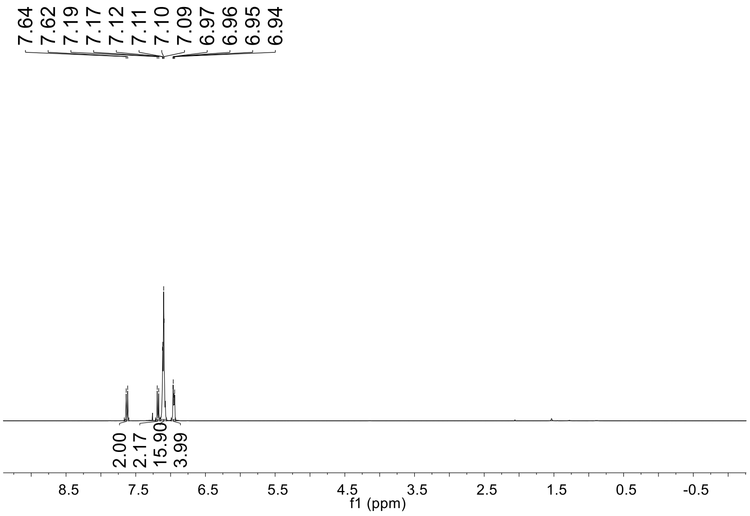

Figure S25. ^{1}H NMR of TPM-Cl ($CDCl_3$, 293 K).

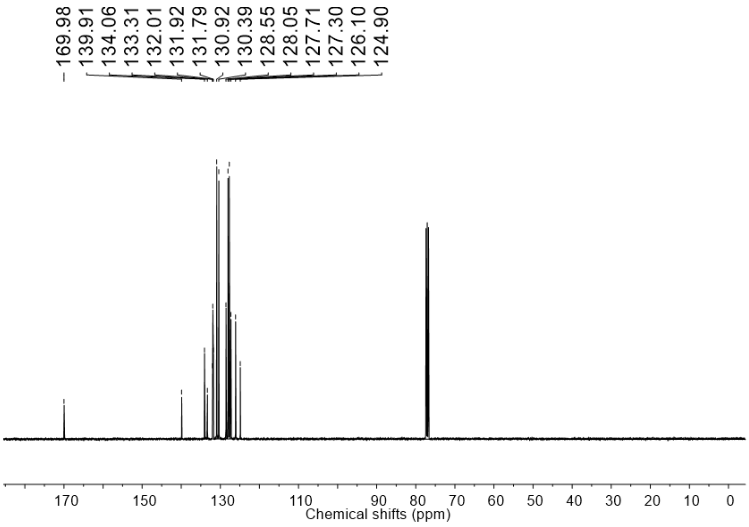

Figure S26. ^{13}C NMR of TPM-Cl ($CDCl_3$, 293 K).

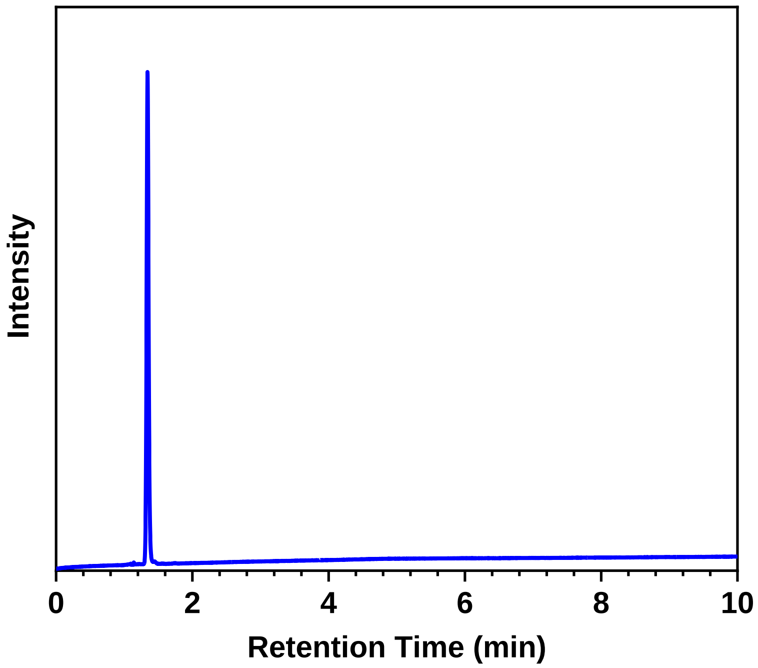

Figure S27. HPLC curve of TPM using acetonitrile as eluent at 40℃.

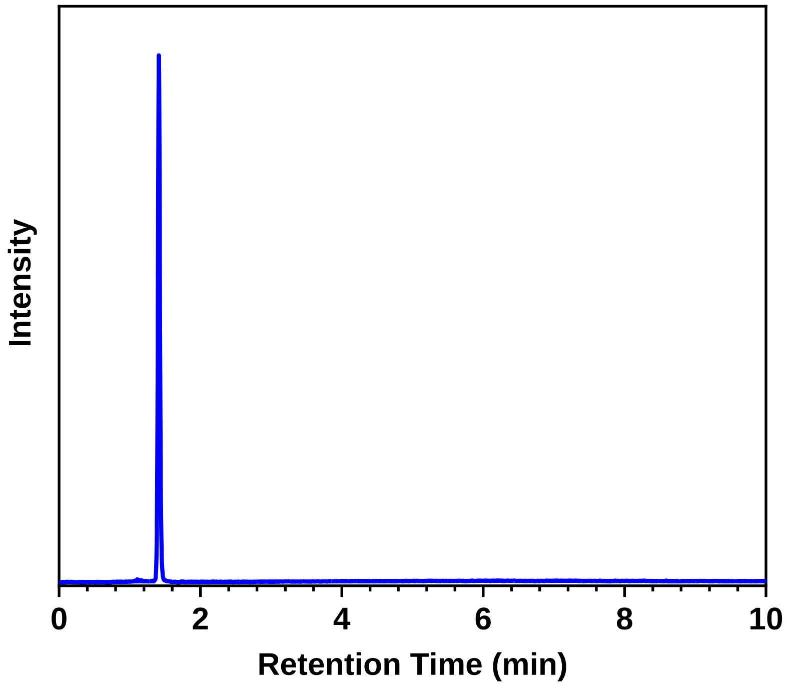

Figure S28. HPLC curve of TPM-Cl using acetonitrile as eluent at 40℃.
