# Supplementary material for: Rational design of pyrrole derivatives with aggregation-induced phosphorescence characteristics for time-resolved and two-photon luminescence imaging
Source: Nat Commun. 2021 Aug 12;12:4883. doi: 10.1038/s41467-021-25174-6 (PMC8361132; doi:10.1038/s41467-021-25174-6)
Supplement: Supplementary file 3 — Reporting Summary [file 41467_2021_25174_MOESM3_ESM.pdf]

nature research

Corresponding author(s): Zhengxu Cai

Last updated by author(s): Jul 7, 2021

Reporting Summary

Nature Research wishes to improve the reproducibility of the work that we publish. This form provides structure for consistency and transparency in reporting. For further information on Nature Research policies, see our Editorial Policies and the Editorial Policy Checklist.

Statistics

For all statistical analyses, confirm that the following items are present in the figure legend, table legend, main text, or Methods section.

n/a	Confirmed	
☐	☒	The exact sample size (n) for each experimental group/condition, given as a discrete number and unit of measurement
☐	☒	A statement on whether measurements were taken from distinct samples or whether the same sample was measured repeatedly
☒	☐	The statistical test(s) used AND whether they are one- or two-sided<br>*Only common tests should be described solely by name; describe more complex techniques in the Methods section.*
☒	☐	A description of all covariates tested
☐	☒	A description of any assumptions or corrections, such as tests of normality and adjustment for multiple comparisons
☐	☒	A full description of the statistical parameters including central tendency (e.g. means) or other basic estimates (e.g. regression coefficient) AND variation (e.g. standard deviation) or associated estimates of uncertainty (e.g. confidence intervals)
☒	☐	For null hypothesis testing, the test statistic (e.g. F, t, r) with confidence intervals, effect sizes, degrees of freedom and P value noted<br>*Give P values as exact values whenever suitable.*
☒	☐	For Bayesian analysis, information on the choice of priors and Markov chain Monte Carlo settings
☒	☐	For hierarchical and complex designs, identification of the appropriate level for tests and full reporting of outcomes
☒	☐	Estimates of effect sizes (e.g. Cohen's d, Pearson's r), indicating how they were calculated

Our web collection on statistics for biologists contains articles on many of the points above.

Software and code

Policy information about availability of computer code

Data collection	TopSpin 3.2, UVWin 5.1.0, FL Solutions 4.2, ZPW388 2.18.1008, Digital Micrograph 2.02.800.0, HitachiH-7650B, Rigaku AFC-10/Saturn 724 +CCD diffractometer with graphite-monochromated Mo Kα radiation (λ=0.71073 Å), Fluorescence lifetime images were taken by ISS Q2 confocal laser scanning system coupled to a Nikon TE2000, IVIS Spectrum CT In Vivo Imaging System
Data analysis	Microsoft Excel 2013, Origin 9.0, ChemOffice 2017, SHELXL-2015 and Olex2 software, Mercury 2.2, Gaussian 09, ORCA 4. 2. 1, Image J 1.52, NIS Viewer 4.50, ROI analysis on Living Imaging

For manuscripts utilizing custom algorithms or software that are central to the research but not yet described in published literature, software must be made available to editors and reviewers. We strongly encourage code deposition in a community repository (e.g. GitHub). See the Nature Research guidelines for submitting code & software for further information.

Data

Policy information about availability of data

All manuscripts must include a data availability statement. This statement should provide the following information, where applicable:

- Accession codes, unique identifiers, or web links for publicly available datasets
- A list of figures that have associated raw data
- A description of any restrictions on data availability

The authors state that the data supporting the results of this study are available in this paper and its supplementary materials. Extra data are available from the corresponding authors upon reasonable request. The data generated in this study have been deposited in the Figshare database: https://doi.org/10.6084/m9.figshare.14913162.v1. The X-ray crystallographic coordinates for structures reported in this study have been deposited at the Cambridge Crystallographic Data Centre (CCDC), under deposition numbers 1975269 and 1975270. These data can be obtained free of charge from The Cambridge Crystallographic Data Centre via www.ccdc.cam.ac.uk/data_request/cif.

Field-specific reporting

Please select the one below that is the best fit for your research. If you are not sure, read the appropriate sections before making your selection.

☒ Life sciences ☐ Behavioural & social sciences ☐ Ecological, evolutionary & environmental sciences

For a reference copy of the document with all sections, see nature.com/documents/nr-reporting-summary-flat.pdf

Life sciences study design

All studies must disclose on these points even when the disclosure is negative.

Sample size	No statistical methods were used to predetermine sample size. For Figure 4 and Figure 5, sample sizes of cells were selected empirically from our previous experimental experience and literatures in the field with similar assays (Angew. Chem. Int. Ed. 2019, 58, 3834; Angew. Chem. Int. Ed. 2020, 59, 8435; J. Am. Chem. Soc. 2019, 141, 5045–5050). For Figure 6, sample sizes of cells were selected empirically based on literatures in the field with similar assays (Nat. Commun. 2019 10, 2111; Nat. Commun.2018, 9, 2963; Nat. Commun. 2018,9, 840). n= 3 mice per group is sufficient to detect meaningful biological difference with good reproducibility.
Data exclusions	No data were exclusion from the analyses.
Replication	All attempts to repeat the experiment (either 3 or 6 independent experiments were repeated when necessary) were successful.
Randomization	No randomization was performed since no experimental groups were used.
Blinding	Blinding is not relevant for this study because it is not a comparative study. For imaging studies of cell and mice, there was no blinding in this study as we did not have different treatment/experimental groups and therefore was not relevant. For other experiments, the investigators were not blinded to the identities of the samples because treatments and data collection were performed by the same people.

Reporting for specific materials, systems and methods

We require information from authors about some types of materials, experimental systems and methods used in many studies. Here, indicate whether each material, system or method listed is relevant to your study. If you are not sure if a list item applies to your research, read the appropriate section before selecting a response.

Materials & experimental systems

n/a	Involved in the study	
☒	☐	Antibodies
☐	☒	Eukaryotic cell lines
☒	☐	Palaeontology and archaeology
☐	☒	Animals and other organisms
☒	☐	Human research participants
☒	☐	Clinical data
☒	☐	Dual use research of concern

Methods

n/a	Involved in the study	
☒	☐	ChIP-seq
☒	☐	Flow cytometry
☒	☐	MRI-based neuroimaging

Eukaryotic cell lines

Policy information about cell lines

Cell line source(s)	HeLa cells were obtained in the present study from National Infrastructure of cell line Resource (NICR).
Authentication	HeLa cells were obtained in the present study from Peking University. None of the cells were authenticated by ourselves.
Mycoplasma contamination	HeLa cells were not tested for mycoplasma contamination.
Commonly misidentified lines (See ICLAC register)	No commonly misidentified cell lines were used in the study.

Animals and other organisms

Policy information about studies involving animals; ARRIVE guidelines recommended for reporting animal research

Laboratory animals	The following strain of mice were used: Balb/c nude mice, 6-weeks-old female mice were used in the experiments. Feeding conditions: all the animals were submitted to controlled temperature conditions (22~26 oC), humidity (50~60%) and light (12 h light/12 h dark, 15~20 LX). They had access to water and food ad libitum in barrier system of Beijing Institute of Technology. (SYXK (Jing) 20170013)

Wild animals	The study did not involve wild animals.
Field-collected samples	The study did not involve Field-collected samples.
Ethics oversight	All experiments were approved by Animal Ethics Committee of Beijing Institute of Technology. The accreditation number is BIT-EC-SCXK(Jing) 2019-0010-M-2020019 promulgated by Animal Ethics Committee of Beijing Institute of Technology.

Note that full information on the approval of the study protocol must also be provided in the manuscript.
